# CHRONIC ILLNESSES AND FATIGUE IN OLDER INDIVIDUALS: A LITERATURE REVIEW

**DOI:** 10.1093/geroni/igz038.1164

**Published:** 2019-11-08

**Authors:** Maral Torossian, Cynthia Jacelon

**Affiliations:** University of Massachusetts Amherst, Amherst, Massachusetts, United States

## Abstract

In the United states, 60% of adults have one chronic disease and 40% have at least two chronic diseases. Fatigue is a commonly reported symptom in individuals with chronic illnesses, the prevalence of which ranges between 40-74%. It is associated with multiple risk factors and has a tremendous impact on quality of life, social functioning, mood, motivation and cognition. Despite its high prevalence, the relationship between fatigue and chronic illness has not been well explored. Accordingly, the focus of this synthesis of literature is to explore fatigue-associated factors and their relation to chronic disease. The databases searched were CINAHL, PubMed, PsychInfo and Web of Science, where the following keywords were used: “Chronic disease” OR “Chronic illness” OR “Chronic conditions”, “Fatigue”, “Elderly” OR “Older adults” OR “Seniors” OR “Geriatrics”. The synthesis resulted in four themes: understanding the concept of fatigue, factors related to fatigue, activity and fatigue, and self-management of fatigue. There were some inconsistencies in the findings among research studies which were addressed, in addition to the strengths and weaknesses of some of the fatigue measurement scales used. This literature review integrates findings about fatigue in chronic illnesses in various aspects, in the population of individuals who are of 65 age or older. The four emerged themes are of value to individuals with similar characteristics as the selected population, as well as to health care providers and researchers who may address the inconsistent findings and provide a strong evidence for best practice.

